# Context-Dependent Parental Effects on Clonal Offspring Performance

**DOI:** 10.3389/fpls.2018.01824

**Published:** 2018-12-06

**Authors:** Bi-Cheng Dong, Mark van Kleunen, Fei-Hai Yu

**Affiliations:** ^1^School of Nature Conservation, Beijing Forestry University, Beijing, China; ^2^Ecology, Biology Department, University of Konstanz, Konstanz, Germany; ^3^Zhejiang Provincial Key Laboratory of Plant Evolutionary Ecology and Conservation, Taizhou University, Taizhou, China; ^4^Institute of Wetland Ecology & Clone Ecology, Taizhou University, Taizhou, China

**Keywords:** *Alternanthera philoxeroides*, clonal plant, individual and whole-generation levels, parental environmental effect, soil nutrients, vegetative offspring

## Abstract

Parental environments may potentially affect offspring fitness, and the expression of such parental effects may depend on offspring environments and on whether one considers an individual offspring or all offspring of a parent. Using a well-studied clonal herb, *Alternanthera philoxeroides*, we first grew parent plants in high and low soil-nutrient conditions and obtained 1st generation clonal offspring from these two environments. Then we grew offspring of these two types of 1st generation clonal offspring also in high and low nutrient conditions. We measured and analyzed mean performance and summed performance of the four types of 2nd generation clonal offspring. High nutrient availability of parental environments markedly increased both mean performance (i.e., the average fitness measure across all individual offspring produced by a parent) and summed performance (i.e., the sum of the fitness measure of all offspring produced by a parent) of the 2nd generation clonal offspring. The positive parental effects on summed performance of the 2nd generation clonal offspring were stronger when the 1st generation clonal offspring grew in the high instead of the low nutrient conditions, but the positive parental effects on their mean performance did not depend on the nutrient environments of the 1st generation clonal offspring. The results provide novel evidence that parental environmental effects persist across vegetative generations and strongly depend on offspring environments and levels of plants.

## Introduction

Vegetative reproduction is a life-history trait that contributes to the wide distribution of clonal plants in natural habitats (Sosnová et al., 2011). Some clonal plants occupying large geographical areas exhibit distinct phenotypes that are in some cases derived from only one genotype or several closely related genotypes (Poulin et al., 2005; Barrett et al., 2008; Gao et al., 2010; Zhang et al., 2010). Clonal (vegetative) offspring ramets are repeatedly produced by parent ramets during the life cycle of clonal plants, and environmental effects experienced by parents may influence performance of clonal offspring. Such parental (environmental) effects have been increasingly considered an important life-history property, acting as an environmental link across generations and influencing the rapid adaptation of offspring to new environments (Schwaegerle et al., 2000; Donohue, 2009; Mousseau et al., 2009; Latzel and Klimešová, 2010; González et al., 2016).

Like genetic effects, parental effects have ecological and evolutionary significance, especially when they can pre-adapt offspring to local conditions that the parents experienced, and which the offspring are also likely to experience (Pigliucci, 2005). Parental effects may modify propagule size to match offspring environments if they are predictable (Allen et al., 2008; Charpentier et al., 2012; Huber et al., 2014). For instance, plants in favorable habitats may produce larger but fewer seeds (or clonal offspring) to shorten time to establishment, resulting in an early competitive advantage in the next generation. By contrast, plants in unfavorable habitats may produce smaller but more offspring to potentially increase offspring dispersal away from the unfavorable habitat but at the cost of individual offspring fitness (Dong et al., 2012; Wang et al., 2014). To achieve a long-term fitness benefit, parental effects may also trigger phenotypic similarity between parents and offspring. For instance, drought-stressed plants may develop longer root systems and produce sexual offspring that also develop longer root systems (Herman et al., 2012), and plants exposed to insect herbivory produce sexual offspring with a strong herbivory-resistant phenotype (Agrawal, 1999, 2002). While many studies have tested parental effects on performance of sexual offspring, few have tested those on performance of clonal offspring and thus little is known about whether parental effects can persist across clonal generations (Latzel and Klimešová, 2010; Huber et al., 2014; González et al., 2016).

**Figure 1:**
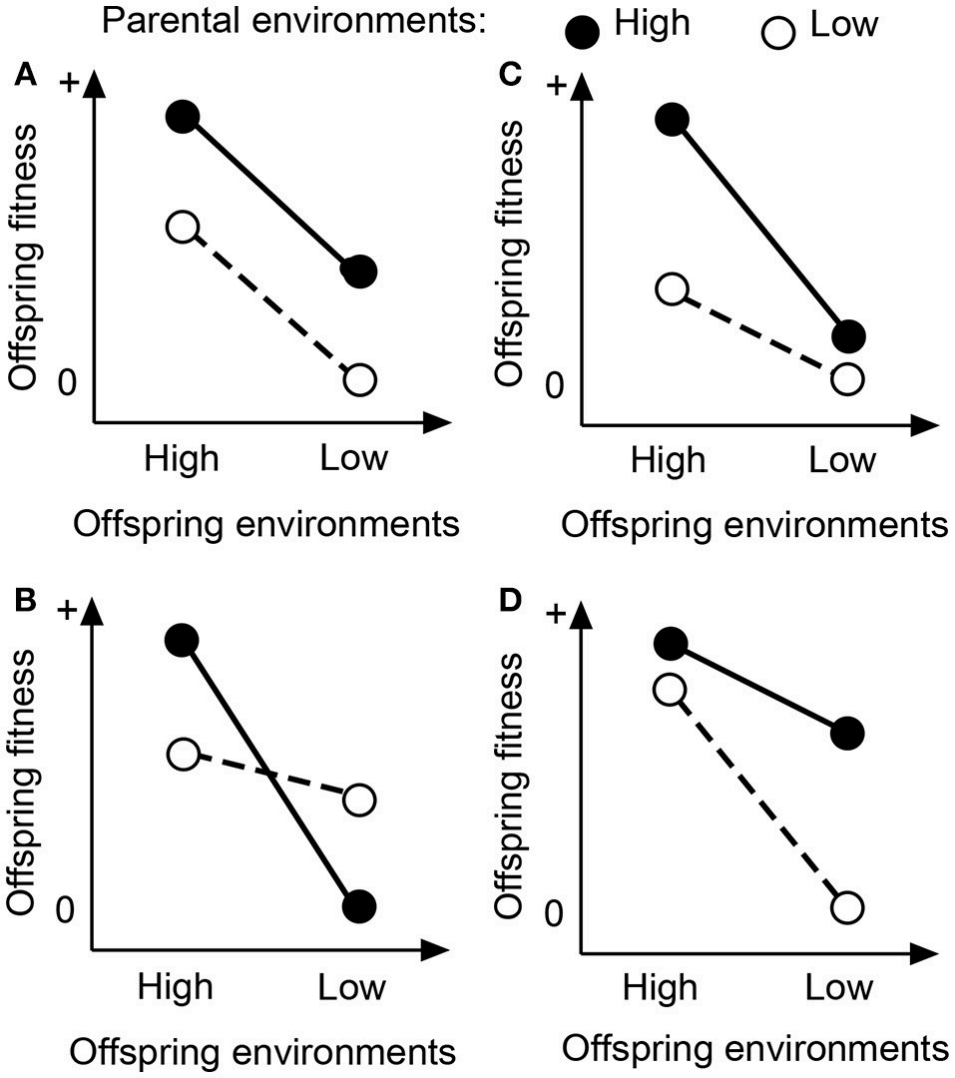
Schematic diagram for parental environmental effects that are not related to offspring environments **(A)**, or that are related to offspring environments **(B–D)**. See the text for details.

Parental effects on offspring fitness can be categorized into four predictable scenarios (Figure 1). In the predictable scenarios, parental effects are assumed to be caused by two types of parental environments, i.e., favorable and unfavorable environments. First, parental effects are independent of offspring environments (Figure 1A; Schwaegerle et al., 2000; Dong et al., 2017, 2018), i.e., offspring of parent plants grown in favorable environments always perform better than offspring of parents grown in unfavorable environments (Uller et al., 2013; Engqvist and Reinhold, 2016). Second, parental effects are context-dependent and adaptive (Figure 1B). Parental effects are advantageous if offspring grow in an environment similar to the one that their parents have encountered (Mousseau and Fox, 1998; Galloway, 2005), and disadvantageous if they grow in an environment dissimilar to the one that their parents have encountered (Dyer et al., 2010). Third, parental effects are advantageous only when offspring grow under favorable environments (Figure 1C). In *Plantago lanceolata*, for example, offspring of parents growing in nutrient-rich soils accumulated more carbohydrates in roots than offspring of parents growing in nutrient-poor soils (Latzel et al., 2014). However, such parental effects were detected only for offspring growing in nutrient-rich soils, and not for offspring in nutrient-poor soils (Latzel et al., 2014). Fourth, parental effects are advantageous only when offspring grow under unfavorable environments (Figure 1D). In animals, for instance, the positive effect of egg size (an indication of maternal nutritional provisioning) is often more pronounced in stressful environments (Fox, 2000; Dziminski and Roberts, 2006).

Although parental effects have been increasingly documented at the individual level (i.e., mean fitness of individual offspring; Huber et al., 2014; González et al., 2016; Groot et al., 2016), they have rarely been explored at the level of the whole offspring generation (i.e., the sum of the fitness measure of all offspring produced by a parent during, e.g., one growing season; Beckerman et al., 2002; Plaistow and Benton, 2009; Molofsky et al., 2014). From an offspring-generation perspective, parental environments may interact with, e.g., offspring survival, size and number, so that the pattern of parental effects at the offspring-generation level is more complex and unpredictable than that at the individual level (Crone, 1997; Charpentier et al., 2012). For instance, due to a potential trade-off between offspring size and number (Stuefer et al., 2010; Dong et al., 2012; Wang et al., 2014), parental effects that are adaptive at individual offspring level may not necessarily be so when fitness of all offspring of a parent are considered (i.e., at the level of the whole offspring generation), and *vice versa*. Given that parental effects have an impact on performance of the offspring generation, they may play an important role in population dynamics (Molofsky et al., 2014). Therefore, it is important to understand parental effects also at the offspring-generation level.

We investigated effects of nutrient environments experienced by parent plants on performance of clonal offspring of a well-studied clonal herb, *Alternanthera philoxeroides*, both at the level of individual offspring and offspring generation. Specifically, we tested the following hypotheses. (1) Parental nutrient effects can persist across vegetative generations in clonal species. One prediction is that clonal offspring produced by parent plants subjected to high soil nutrients will perform better than do the offspring produced by parent plants subjected to low soil nutrients, since providing parent plants with high soil nutrients may allow them to produce high-quality clonal offspring. (2) The magnitude of parental nutrient effects depends on the nutrient environments of clonal offspring. One prediction is that providing clonal offspring with high nutrient levels amplifies parental effect as shown in Figure 1C. (3) Parental nutrient effects at the offspring-generation level are inconsistent with the effects at the individual-offspring level. This is because parental effects at the individual level are determined only by average offspring size, while parental effects at the generation level are determined jointly by the survival, size and number of offspring.

## Materials and Methods

### Study Species and Plant Material

*Alternanthera philoxeroides* (Mart.) Griseb. is a creeping perennial herb of the Amaranthaceae family, native to South America (Holm et al., 1997). It is listed as one of the most noxious invasive plants in China and other regions around the world (Julien et al., 1995; Sainty et al., 1998). In China, for lack of viable seeds, *A. philoxeroides* mainly relies on clonal growth by producing creeping stems and/or root fragments to achieve offspring recruitment (Wang et al., 2008, 2009). Each stem node of *A. philoxeroides* is considered an asexual individual (i.e., ramet), because it has the potential to root and develop into a physiologically independent plant (Dong et al., 2010, 2012). This species can rapidly disperse and colonize both aquatic and terrestrial habitats, causing severe economic and environmental problems (Wang et al., 2008, 2009).

For our study, original plants of *A. philoxeroides* were collected on 18–19 May 2011, from several locations in a riparian agricultural area in Zhejiang province (28.87° N, 121.01° E), in the south of China. The sampling site did not belong to any farms or national parks, so that we did not need any relevant permissions for collecting plant samples. To reduced potential phenotypic differences among the plants due to variation in parental environments, the plants had been propagated vegetatively for 4 years in a heated greenhouse at Forest Science Co., Ltd., of Beijing Forestry University. In China and Australia, *A. philoxeroides* does not produce viable seeds (Sainty et al., 1998; Zhu et al., 2015). Studies using molecular markers showed that populations sampled in South China derived from a single genotype (Xu et al., 2003; Wang et al., 2005; Li and Ye, 2006). Thus, it is very likely that the plants we collected and their clonal offspring share the same genotype.

### Experimental Design

**Figure 2:**
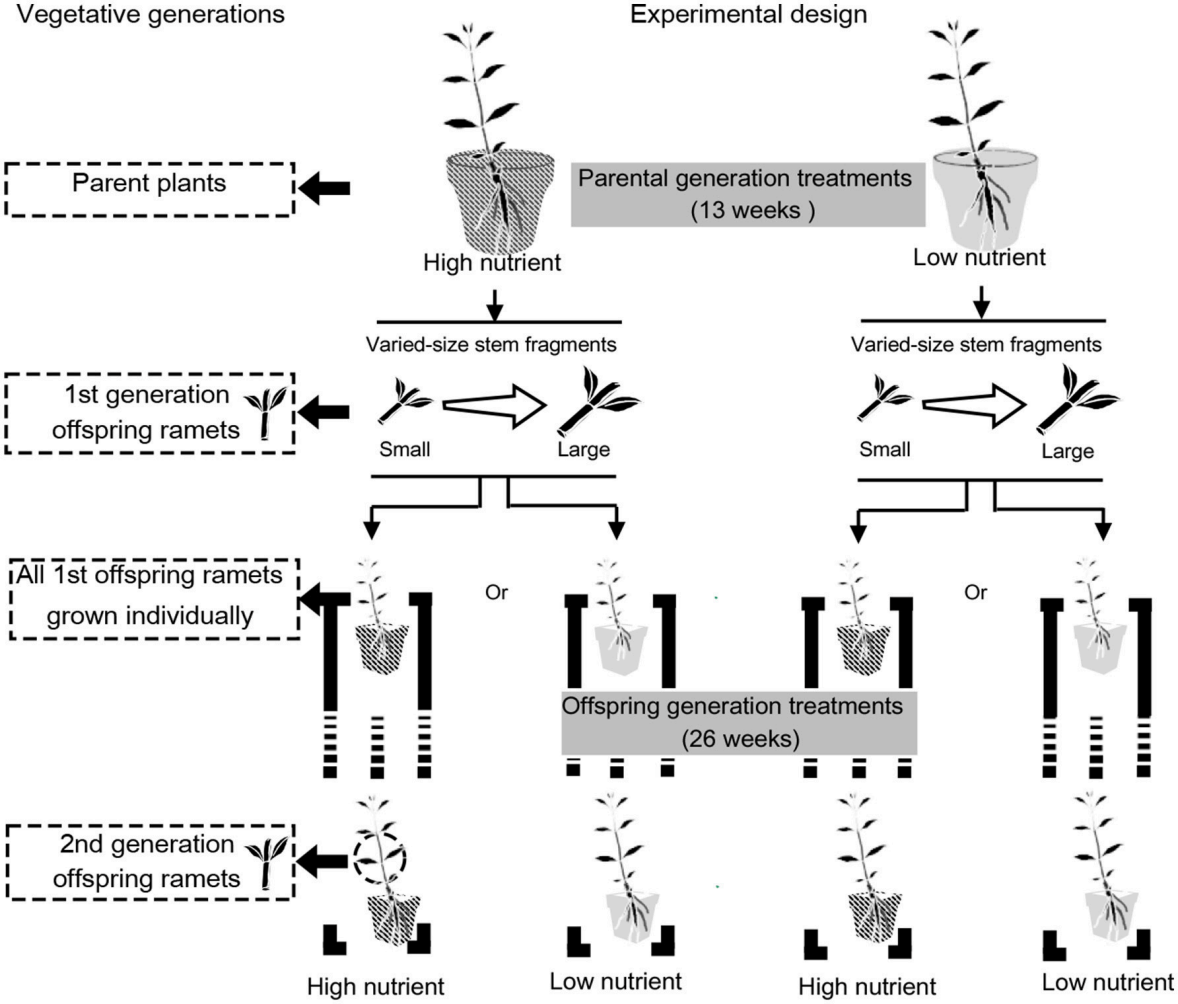
Experimental design. The experiment consisted of two steps. First, we grew parent plants in high and low soil nutrient conditions and obtained two types of the 1st generation clonal offspring ramets. Then we grew the 1st generation clonal offspring ramets of each type also in high and low nutrient conditions and measured the 2nd generation clonal offspring ramets.

The experiment consisted of two steps (Figure 2). In brief, we first grew 42 parent plants each with a stem of about 15 cm long in high and low soil nutrient conditions (i.e., 21 replicates per parental treatment) and obtained seven replicates of two types of the 1st generation clonal offspring ramets for growth measurements and the remaining 14 replicates for subsequent experiment. Then we grew seven replicates of 1st generation clonal offspring ramets of each type also in high and low nutrient conditions and measured the 2nd generation clonal offspring ramets (Figure 2).

In more detail, on 28 June 2014, 100 stem fragments of *A. philoxeroides*, each consisting of one node bearing two opposite leaves and 3-cm-long proximal and distal internodes, were cut off from the stock plants. In the same greenhouse as the pre-cultivation, fragments were grown in planting trays filled with an 1:1 volume mixture of quartz sand (0.5–1.0 mm particle size) and peat (Pindstrup Seedling, Pindstrup Mosebrug A/S, Pindstrup, Denmark). After 3 weeks when most fragments had produced a new stem of ~15 cm long, we selected 60 fragments (plants) of similar sizes. Of the 60 plants, 18 were harvested to measure initial dry mass (mean: 111.5 mg; 95% confidence interval: 100.1–122.4 mg; *N* = 18). The remaining 42 plants were used as parent plants, and were transplanted into pots that were 14 cm in diameter and 12 cm in depth and filled with the soil mixture described above.

We randomly assigned the 42 parent plants to two soil nutrient treatments, and thus each treatment had 21 replicates. For the high-nutrient treatment, 2 g L^−1^ of slow-release fertilizer (16 N: 9 P: 12 K: 2 Mg; Osmocote Standard, Scotts, Marysville, Ohio, USA) was mixed into the soil of each pot. For the low-nutrient treatment, no fertilizer was added. The two nutrient treatments are the nutrient conditions commonly experienced by the species. Pots were randomly repositioned once a month to minimize possible effects of environmental heterogeneity in the greenhouse. Tap water was supplied daily to keep the soil moist. The treatments lasted for 13 weeks, during which the mean air temperature (± SE) in the greenhouse was 23.1 ± 0.4°C, as measured by a Hygrochron temperature logger (iButton DS1923; Maxim Integrated Products, USA).

On 18 October 2014, we randomly chose seven replicate plants (parent ramets with clonal offspring ramets) in each treatment and counted the number of offspring ramets. The plants were then subdivided into the aboveground part (the assembly of single-node offspring ramets attached with two opposite leaves and half of both the proximal and distal internodes) and belowground part (roots), and dried at 70°C for 48 h.

For each of the remaining 14 replicate plants in each of the two nutrient treatments, we obtained single-node offspring ramets (a stem node attached with two opposite leaves and a half of proximal and distal internodes) by cutting off the nodes along the newly produced stems of each parent plant. Each of these single-node ramets (i.e., the 1st generation clonal offspring) was labeled to mark its position along the stems produced by the parent plants, and weighed to obtain initial fresh mass. The parent plants in the high-nutrient treatment each produced 10–47 offspring ramets, and those in the low-nutrient treatment each produced 6–19 offspring ramets. Each of the 1st generation offspring ramets taken from seven randomly selected plants of each of the two nutrient treatments was grown in the high nutrient treatment (adding 2 g L^−1^ of slow-release fertilizer to the soil), and each of the 1st generation offspring ramets taken from the remaining seven plants of each of the two nutrient treatments was grown in the low nutrient treatment (no fertilizer added). The soil mixture used for the 1st generation offspring ramets was the same as that for the parent plants, and all offspring ramets taken from one parent plant were grown in different cells (each 4.6 cm long × 4.6 cm wide × 11 cm deep) within the same planting tray, and subjected to one nutrient treatment. There were seven replicate trays for each of the four parent-offspring treatment combinations. Trays were randomly repositioned every month.

The treatments for the 1st generation offspring lasted for 26 weeks, from 18 October 2014 to 18 April 2015. They were conducted in the same greenhouse (mean temperature ± SE was 15.4 ± 0.2°C). At harvest, we recorded the survival status of the originally planted offspring ramets. We counted the number of the 2nd generation ramets originated from each of the 1st generation offspring ramets and also measured biomass of the 2nd generation ramets that originated from each of the 1st generation offspring ramets by drying them at 70°C for 48 h. Based on these data, we calculated the summed mass and summed number of the 2nd generation ramets produced by all the 1st generation offspring ramets from each parent plant. We also calculated mean mass and mean number of the 2nd generation ramets per 1st generation offspring from each parent plant (summed mass or number of the 2nd generation ramets divided by number of the 1st generation offspring ramets from each parent plant).

### Data Analyses

We used *t*-tests to examine the effects of soil-nutrient treatments on total mass, number of ramets and mean ramet mass (shoot mass divided by number of ramets) of the 1st generation offspring (i.e., the ramets produced by the parent plants). We used two-way ANOVAs to test the effects of parental nutrient conditions (fixed effect), offspring nutrient conditions (fixed effect) and their interaction (fixed effect) on performance of *A. philoxeroides* at both the offspring generation level (summed mass and summed number of the 2nd generation ramets produced by all the 1st generation offspring from one parent plant) and the individual level (mean mass and mean number of the 2nd generation ramets across the 1st generation offspring ramets from a parent plant). We also used two-way ANOVAs to test the effects of parental and offspring nutrient treatments on initial fresh mass and number of the surviving 1st generation offspring ramets as well as survival rate of the ramets. These data met the assumptions of homoscedasticity and normality. The analyses were conducted using SPSS 22.0 (SPSS, Chicago, IL, USA).

A three-parameter lognormal distribution $\left(\text{Y}\;={\frac{\text{a}}{\text{X}}}^{*}\exp\left(-0.5^{*}{\left(\frac{\ln\left(\frac{\text{X}}{\text{X0}}\right)}{\text{b}}\right)}^{\text{2}}\right)\right)$ was employed to fit the frequency distribution of initial fresh mass of the pooled 1st generation offspring ramets produced by parent plants grown in the high nutrient or the low nutrient treatments. In the equation, X is the initial mass of each 1st generation offspring ramet; X_0_, a, and b are the location parameter, the scale parameter, and the shape parameter of distribution, respectively. The regression analyses were performed using Sigmaplot 12.5 (Systat Software Inc., San Jose, CA, USA).

## Results

### Performance of the Parental Generation

**Figure 3:**
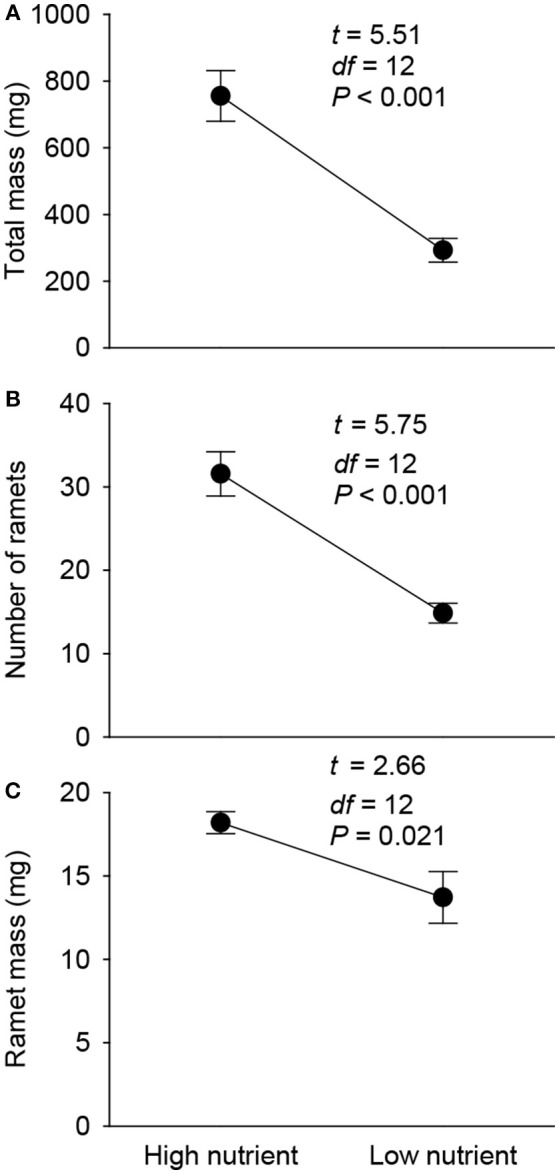
Effects of parent nutrient treatment on total mass **(A)**, number of ramets **(B)**, and mean ramet mass **(C)** of the 1st generation offspring of *Alternanthera philoxeroides*. Means + SE and *t, df*, and *P* values of *t*-test are given.

Total mass, number of ramets and mean ramet mass of the 1st generation offspring ramets produced by the parent plants were all significantly lower in the low nutrient than in the high nutrient treatment (Figure 3).

### Performance of the Offspring Generation

**Figure 4:**
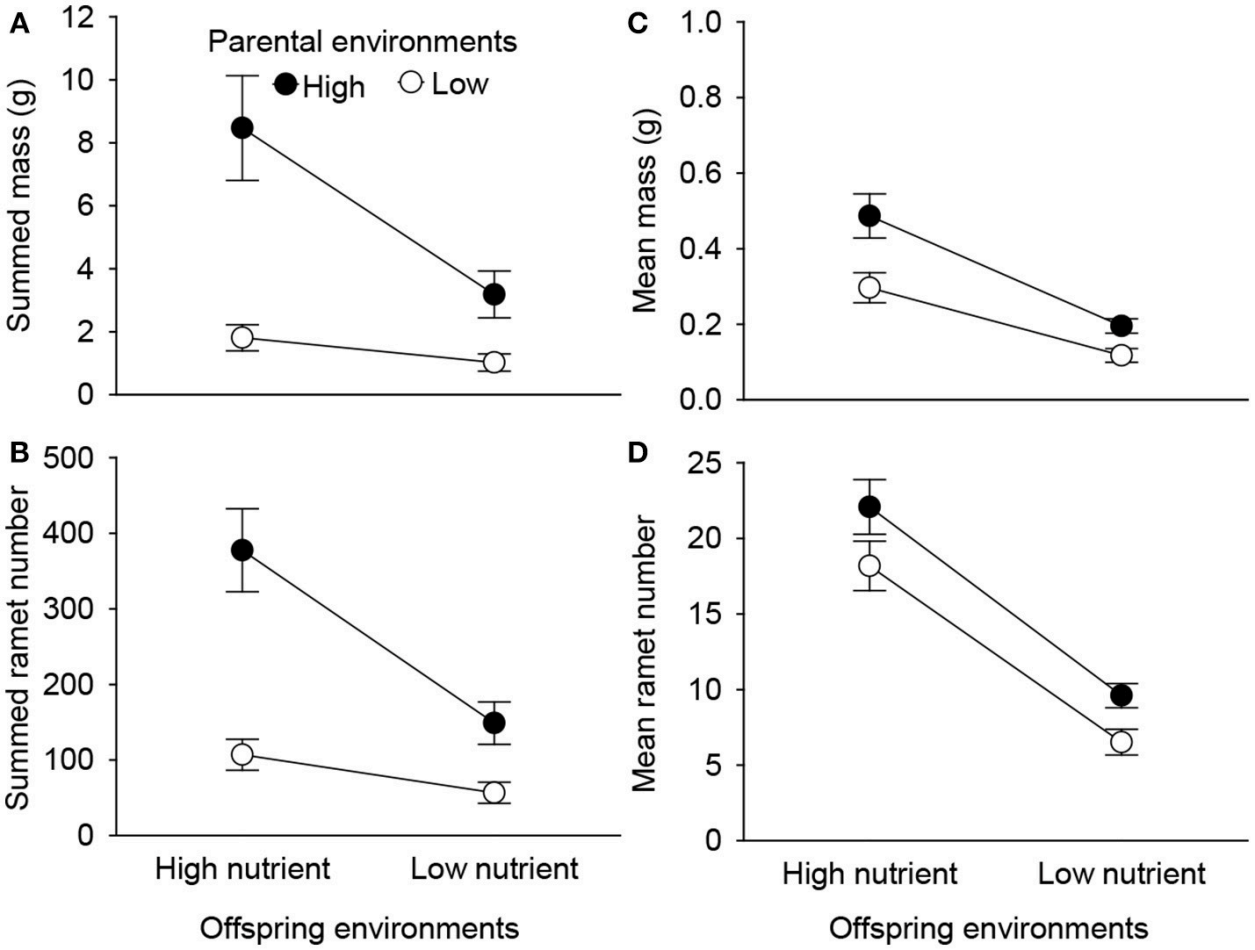
Effects of parent and offspring (1st generation) nutrient treatments on summed mass **(A)**, summed ramet number **(B)**, mean mass **(C)**, and mean ramet number **(D)** of the 2nd generation offspring of *Alternanthera philoxeroides* across the 1st generation offspring of a parent plant. Means + SE are given.

Summed mass and number of the 2nd generation offspring ramets were significantly affected by parental and offspring environments, as well as by their interaction (Table 1, Figure 4). High nutrient availability of parental environments markedly increased summed performance of all 2nd generation offspring produced in the offspring generation (Figures 4A,B). However, these positive parental effects were stronger when the 1st generation offspring grew in the high than in the low nutrient conditions (Figures 4A,B). By contrast, mean mass and mean number of the 2nd generation offspring ramets were independently affected by parental and offspring environments (Table 1). High nutrient availability of parental and offspring environments both increased mean mass and mean number of the 2nd generation offspring ramets, but the positive parental effects did not depend on the nutrient environments of the 1st generation offspring (Figures 4C,D).

**Table 1:** Effects of parent and offspring (1st generation) nutrient treatments on summed and mean performance of the 2nd generation offspring across the 1st generation offspring of a parent plant.

**Measure**	**Parent (P)**		**Offspring (O)**		**P** **×** **O**	

	***F***_**(1, 24)**_	***P***	***F***_**(1, 24)**_	***P***	***F***_**(1, 24)**_	***P***
Summed mass	**21.83**	**<0.001**	**10.34**	**0.004**	**5.67**	**0.026**
Summed ramet number	**29.83**	**<0.001**	**17.65**	**<0.001**	**7.20**	**0.013**
Mean mass	**12.62**	**0.002**	**38.77**	**<0.001**	2.20	0.151
Mean ramet number	**6.68**	**0.016**	**79.83**	**<0.001**	0.09	0.765

*Degrees of freedom (df), F, and P of ANOVA are given. Values for which P < 0.05 are shown in bold*.

**Figure 5:**
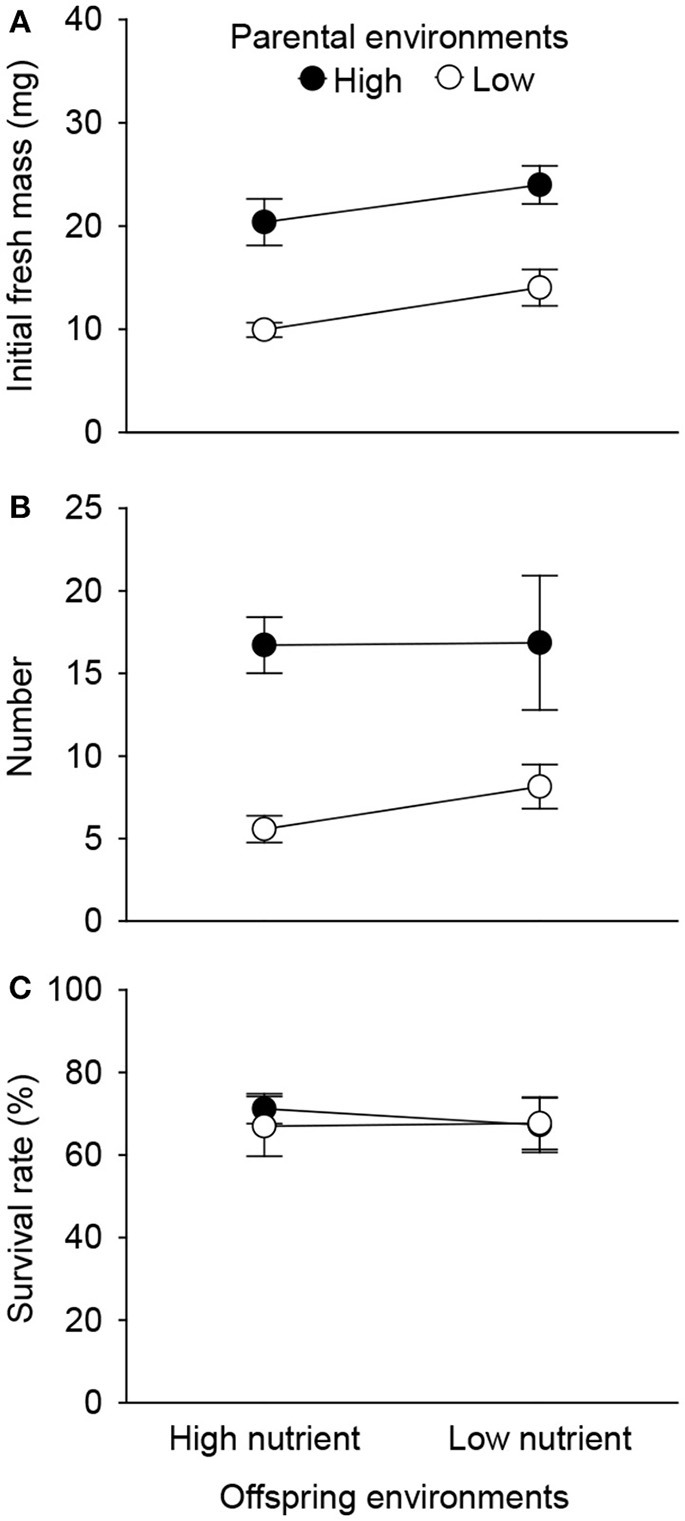
Effects of parent and offspring (1st generation) nutrient treatments on initial fresh mass **(A)** and number **(B)** of the surviving 1st generation offspring ramets, and survival rate **(C)**. Means + SE are given.

Initial fresh mass of the surviving 1st generation offspring was independently affected by parental and offspring environments (Table 2). High nutrient availability of parental environments increased initial fresh mass, and high nutrient availability of offspring environments allowed the smaller 1st generation offspring ramets to survive during the experiment (Figure 5A). The number of the surviving 1st generation offspring ramets was only affected by parental environments, rather than by offspring environments (Table 2). High nutrient availability of the parental environments increased number of the surviving 1st generation offspring ramets (Figure 5B). By contrast, survival rate of the 1st generation offspring was affected by neither parental nor offspring environments (Table 2, Figure 5C).

**Table 2:** Effects of parent and offspring (1st generation) nutrient treatments on initial fresh mass and number of the surviving 1st generation offspring ramets and survival rate.

**Measure**	**Parent (P)**		**Offspring (O)**		**P** **×** **O**	

	***F***_**(1, 24)**_	***P***	***F***_**(1, 24)**_	***P***	***F***_**(1, 24)**_	***P***
Initial fresh mass	**34.31**	**<0.001**	**4.88**	**0.037**	0.02	0.894
Number	**18.06**	**<0.001**	0.34	0.567	0.27	0.608
Survival rate	0.10	0.758	0.07	0.791	0.14	0.705

*Degrees of freedom (df), F, and P of ANOVA are given. Values for which P < 0.05 are shown in bold*.

**Figure A1:**
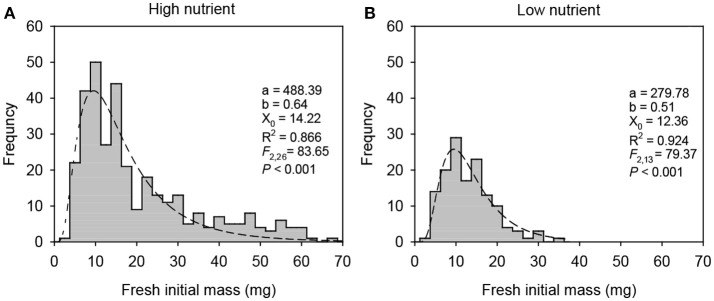
The frequency distribution of initial fresh mass of the 1st generation offspring ramets produced by parent plants grown in **(A)** the high nutrient or **(B)** the low nutrient treatments. Each frequency distribution of the pooled 1st generation ramets subject to one of parent nutrient treatments fits well to the lognormal distribution. The equation of 3-parameter lognormal distribution is $\left(\text{Y}\;=\frac{\text{a}}{\text{X}}*\exp\left(-0.5*{\left(\frac{\ln\left(\frac{\text{X}}{\text{X0}}\right)}{\text{b}}\right)}^{2}\right)\right)$, where X is the initial mass of each 1st generation offspring ramet; X_0_, a, and b are the location parameter, the scale parameter, and the shape parameter of distribution, respectively. The fitted parameters of size distribution (a, b, and X_0_), R^2^, *F* and *P* values are given.

Frequency distribution of the pooled 1st generation offspring ramets subjected to each of parental nutrient treatments fitted well to the lognormal distribution (Figure A1). The distribution of 1st generation offspring ramets produced by parent plants growing in the high nutrient treatment was positively skewed [spanning a broad range of 2.5–66.1 mg; *R*^2^ = 0.886, *F*_(2, 26)_ = 83.65, *P* < 0.001]. By contrast, the distribution of 1st generation offspring ramets produced by parent plants growing in the low nutrient condition tended to be platykurtic (flat) and symmetrical [spanning a narrow range of 1.8–34.3 mg; *R*^2^ = 0.924, *F*_(2, 15)_ = 79.37, *P* < 0.001; Figure A1].

## Discussion

For the parent generation of *A. philoxeroides*, limited soil nutrients reduced biomass accumulation and new ramet production by ~50% and mean ramet mass (or vegetative offspring size) by 20%. These results were consistent with the negative responses of *A*. *philoxeroides* to low resource availability (e.g., Li et al., 2014). Interestingly, parental nutrient environments exerted a strong effect on performance across vegetative generations. One apparent reason is that the offspring from parents growing in the nutrient-rich environment were relatively larger, having about 2-fold greater initial mass than did offspring taken from parents growing in the nutrient-poor environment. Such a size advantage of offspring benefited the subsequent growth of offspring both in the high and in the low nutrient environment. The variation in offspring size, and the corresponding provisioning of internal resources (e.g., non-structural carbohydrates and nitrogen) may be one of potential mechanisms that triggered the observed variation in fitness between offspring ramets taken from parents growing in contrasting habitats (Herman and Sultan, 2011; Latzel et al., 2014).

We also found that the magnitude of parental effects depended on the environment of the offspring, i.e., the positive effect of the parental high-nutrient treatment was amplified when the offspring were also in a high-nutrient environment (Figure 1C). To some degree, parental effects could facilitate the pre-adaptation of offspring of *A. philoxeroides* to their parental environment by modifying offspring size, thereby helping to gradually accumulate a size advantage over previous generations in favorable habitats. Such a life history may possibly contribute to the abundance and invasiveness of *A. philoxeroides* in the environments where resource availability is high, e.g., crop fields and irrigation ditches (Pan et al., 2006). The ecological significance of parental effects have also been reported in many sexually propagated species (Miao and Primack, 1991; Miao et al., 1991; Herman et al., 2012; Jacobs and Lesmeister, 2012; Latzel et al., 2014). For example, parental effects could maximize biomass and root carbohydrate storage accumulation in *P. lanceolata*, seedling yield in *Campanulastrum americanum* and drought tolerance in *Polygonum persicaria*, when the offspring grew in the environments similar to their parental environments (Galloway and Etterson, 2007; Herman et al., 2012; Latzel et al., 2014).

While positive parental nutrient effects were detected at both individual and whole-generation levels, the patterns of these parental effects differed. Context-dependent parental effects in *A. philoxeroides* were detected at the offspring generation level (summed performance of the 2nd generation offspring across all the 1st generation offspring ramets from a parent plant), but not with respect to individual ramet performance (mean performance of the 2nd generation offspring across the 1st generation offspring ramets of a parent plant). One possible reason is that parental effects at the offspring-generation scale were jointly influenced by offspring size and offspring number, while parental effects at the individual offspring scale were only determined by mean offspring size (Hopper et al., 2003; Charpentier et al., 2012; Dong et al., 2012). Our results detected that parental nutrient environments significantly altered the survival and production of offspring, so we speculated that such variation in number of surviving 1st generation individuals may facilitate the parental effects on the summed fitness of offspring in the nutrient-rich environment, but contributed less to the parental effects on the summed offspring fitness in the nutrient-poor environment (Schwaegerle et al., 2000). Besides, parent plants growing in the high nutrient condition produced a positively skewed size distribution of offspring ramets, while parent plants growing in the low nutrient condition only produced a relatively platykurtic (flat) and symmetrical size distribution. To some extent, the changes in size distributions of offspring ramets caused by parental environments may potentially result in a difference in parental effects at individual and whole-generation levels. However, the ecological significance of the offspring-size distribution with regard to parental effects should be further explored in future studies.

We thus conclude that parental nutrient effects can persist across clonal generations of *A*. *philoxeroides* in terms of offspring size and number. Such positive parental effects may contribute to the colonization of *A*. *philoxeroides* in resource-rich habitats because parental effects lead to a gradual increase in the size advantage across clonal generations in such habitats (Marshall and Uller, 2007; Dyer et al., 2010; Gao et al., 2010). Our study also provides novel evidence that the ecological significance of parental environmental effects vary at different levels, implying that these effects cannot simply be extrapolated from the individual to the whole-generation level. Apart from the variation in offspring size (or resource provisioning) and offspring number, parental environmental effects may also be closely related to multiple external or internal factors, including morphological and physiological changes (e.g., plant vigor) and epigenetically based variation (e.g., DNA methylation; Bossdorf et al., 2008; Herman and Sultan, 2011; Zhang et al., 2013; Douhovnikoff and Dodd, 2015; Dodd and Douhovnikoff, 2016). Therefore, future studies that integrate morphological, physiological and molecular evidence should be necessary to better understand the mechanisms of parental environmental effects in clonal species.

## Author Contributions

B-CD and F-HY designed the experiment. B-CD performed the experiment. B-CD and F-HY did the statistical analysis. B-CD, MvK, and F-HY wrote the first draft of the manuscript. B-CD and F-HY contributed substantially to the revisions.

### Conflict of Interest Statement

The authors declare that the research was conducted in the absence of any commercial or financial relationships that could be construed as a potential conflict of interest.
